# Lipocalin 2 potentially contributes to tumorigenesis from colitis via IL-6/STAT3/NF-κB signaling pathway

**DOI:** 10.1042/BSR20212418

**Published:** 2022-05-13

**Authors:** Se Lim Kim, Min Woo Shin, Seung Young Seo, Sang Wook Kim

**Affiliations:** 1 Department of Internal Medicine Research Institute of Clinical Medicine, Jeonbuk National University Medical School, Jeonju, Korea; 2 Biomedical Research Institute, Jeonbuk National University Hospital, Jeonbuk National University Medical School, Jeonju, Korea

**Keywords:** Colitis-associated cancer, Colorectal cancer, Interleukin-6, Lipocalin 2, NF-kB, STAT3

## Abstract

Lipocalin (LCN) 2 (LCN2), a member of the lipocalin superfamily, plays an important role in oncogenesis and progression in various types of cancer. However, the role of LCN2 in inflammation-associated cancer remains unknown. Here, we explored the functional role and mechanisms of LCN2 in tumorigenesis using murine colitis-associated cancer (CAC) models and human colorectal cancer (CRC) cells. Using murine CAC models, we found that LCN2 was preferentially expressed in colonic tissues from CAC models compared with tissues from normal mice. *In vitro* results demonstrated that the levels of LCN2 mRNA and protein were markedly up-regulated by interleukin (IL) 6 (IL-6) in human CRC cells. Interestingly, we found LCN2 up-regulation by IL-6 is diminished by nuclear factor-κB (NF-κB) and signal transducer and activator of transcription 3 (STAT3) inhibition using specific inhibitors and small interfering RNA (siRNA). Reporter assay results determined that IL-6 induces LCN2 gene promoter activity under control of NF-κB/STAT3 activation. IL-6-induced LCN2 regulated cell survival and susceptibility of developmental factors to the NF-κB/STAT3 pathway. Taken together, our results highlight the unknown role of LCN2 in CAC progression and suggest that increased LCN2 may serve as an indicator of CRC development in the setting of chronic inflammation.

## Introduction

Inflammatory bowel diseases (IBDs) can be divided into two major disorders: ulcerative colitis and Crohn’s disease [1]. Although colitis-associated colorectal cancer (CAC) accounts for under 5% of all cases of colorectal cancer (CRC), patients with IBDs are at a greater risk conditions of CRC than the general population [2]. Today, CRC accounts for up to 15% of all deaths among IBD patients. Patients with IBD are six times more likely to develop CRC than the general population and have a higher frequency of multiple synchronous CRC [3,4]. Moreover, IBD incidence is highest among younger patients as the average age of CRC in patients with IBD is lower than that of sporadic CRC [5]. Early detection and prevention strategies (such as colonoscopy, mucosal biopsies, and proctocolectomy) for CRC patients with ulcerative colitis have limitations, and therefore, there is increased interest in identifying target genes to reduce the overall risk of CAC.

There is growing evidence that tumors are sustained and promoted by inflammatory signals from the surrounding microenvironment [6]. Production of tumor-promoting cytokines by inflammatory cells that activate transcription factors, such as nuclear factor-κB (NF-κB), signal transducer and activator of transcription 3 (STAT3) and activator protein 1 (AP-1), in pre-malignant cells to affect genes that induce cell proliferation and survival, is a major carcinogenesis mechanism [7]. Among transcription factors, NF-κB and STAT3 are activated in the majority of cancers and these play critical roles in mediating the link between inflammation and cancer development by activating genes that control cell survival, proliferation, invasion, and cytokine production [8,9].

Interleukin (IL) 6 (IL-6) is one of the major proinflammatory cytokine that plays an important role in promoting inflammation and neoplastic transformation. Many studies report that IL-6 is increased and its expression level is correlated with the severity of disease in UC, and IL-6 contributes to colonic tumorigenesis in these patients [8,10–12]. Furthermore, IL-6 strongly promotes the development of CAC in mice, whereas blocking of IL-6 signaling by injection of IL-6 receptor antibody can successfully suppress intestinal inflammation and CAC [13–15]. Therefore, identification of IL-6 signaling is important to better understand the molecular mechanisms of colorectal tumorigenesis, including CAC, and more importantly to identify new therapeutic and preventive molecular targets.

Lipocalins (LCNs) comprise a class of proteins with various biological activities, including immune responses, cell growth and metabolism, iron transport, and prostaglandin synthesis [16]. LCN2, also known as oncogene 24p3 neutrophil gelatinase-associated lipocalin (NGAL), is a 25-kDa secreted glycoprotein that was initially purified from neutrophil granules [17]. LCN2 has binding capabilities for hydrophobic molecules including retinoids, fatty acids, and various steroids [18]. Recent reports have shown systemic up-regulation of LCN2 in IBD murine models and patients with ulcerative colitis, suggesting that LCN2 has potential as a sensitive biomarker for intestinal inflammation [19–23]. During cancer progression, LCN2 expression is regulated in a variety of human epithelial cancers, including breast, ovarian, pancreatic, oral, lung, esophageal and CRC [24–26]. Interestingly, the expression pattern and functional role of LCN2 appear to vary depending on the type of cancer [26,27]. Thus, the expression pattern and functional roles of LCN2 in cancer remain unclear and controversial. In our previous study, we demonstrated that LCN2 is predominantly involved in the early stages of tumorigenesis of CRC, and it is negatively correlated with advanced stage disease and metastasis in CRC patients [28]. However, a deep understanding of the molecular mechanism of LCN2 as a target for CAC is still lacking. A new perspective of LCN2 as a molecular target for CAC would provide new insight into the function of LCN2 in oncogenesis. We performed the present study to investigate the role and potential mechanism of LCN2 in the regulation CAC tumorigenesis, especially in relation to the IL-6 signaling pathway.

## Materials and methods

### Chemicals and reagents

IL-6 was purchased from R&D Systems (Minneapolis, MN). Parthenolide (PT) and stattic were obtained from Sigma-Aldrich (St. Louis, MO). IL-6 was dissolved in 1× phosphate-buffered saline (PBS) to a concentration of 100 μg/ml and stored at −20°C. PT was dissolved in dimethylsulfoxide (DMSO; Sigma–Aldrich) to a concentration of 100 μM and stored at −20°C in the dark. Stattic was also dissolved in DMSO to a concentration of 50 mM at −20°C.

### CAC murine models

Thirty specific pathogen-free mice (Balb/C female mice, 6 weeks old) were purchased from Orient (Seongnam, Korea). Mice were given water and standard rodent food and maintained on a 12/12-h light/dark cycle under specific pathogen-free conditions. Mice were randomly assigned to the normal control and CAC groups. After then, the mice were intraperitoneally injected with 7.4 mg/kg body weight of azoxymethane (AOM, Sigma–Aldrich) dissolved in physiological saline. After 7 days, 2% dextran sulfate sodium (DSS, MP Biomedicals, Santa Ana, CA) was given in their drinking water for 7 days, followed by 2-week consumption of regular water. This cycle was repeated three times. After finishing CAC-related procedures, all mice were killed by cervical dislocation and their body weight was measured. All colons were removed and their lengths were measured by Vernier calipers. Then, the colons were cut open longitudinally and washed with 1× PBS. After gross examination, half of each groups’ specimens were fixed in 10% neutral-buffered formalin for histological staining and immunohistochemistry. The remaining colons were used for ELISA and Western blot analyses. All procedures of animal experiments were reviewed and approved by Jeonbuk National University Animal Care and Use Committee (Approval number: CBNU 2018-001). All laboratory animals were cultivated and worked in Jeonbuk National University Medical School. Animal experiments were performed in strict compliance with European guidelines and regulations on protection of animals used for scientific purposes (EC Directive 2010/63/EU).

### Histological staining and immunohistochemistry

Paraffin-embedded samples were cut into 5-μm-sections, and then Hematoxylin and Eosin (H&E) were performed for light microscopic examination. The sections were immunostained with anti-LCN2 antibody, visualized by appropriate biotin-conjugated secondary antibodies followed by immunoperoxidase detection with Anti-Goat HRP-DAB Cell & Tissue Staining Kit (Cell Signaling Technology, Danvers, MA) and counterstained with Hematoxylin (Sigma–Aldrich, St. Louis, MO). The sections of colon were photographed with a Leica DM750 (Wetzlar, Germany) photomicroscope.

### LCN2 ELISA

Mouse LCN2 ELISA Kits (R&D Systems) were used to measure LCN2 levels in mouse colon tissue lysates per the manufacturer’s instructions. All samples were assayed in duplicate and compared with a standard curve to quantitate expression.

### Cell culture

Human CRC cell lines DLD-1, HT-29, and SW480 were purchased from the American Type Culture Collection (ATCC; Manassas, VA). The cells were cultured in RPMI-1640 (Gibco™, Thermo Fisher Scientific, MA, U.S.A.), including 10% fetal bovine serum (FBS, Gibco™), 100 units penicillin (Gibco™), and 100 units streptomycin (Gibco™) in a humidified 5% CO_2_ environment at 37°C.

### RNA isolation and reverse transcription-PCR

The cells were resolved in a TRIzol reagent (Invitrogen, Eugene, OR) for obtaining total RNA, and cDNA was synthesized with GoScript™ reverse-transcriptase (Promega, Madison, WI) according to the manufacturer’s protocol. GAPDH was used as an internal control gene. The following primer sequences were used: LCN2 5′-TCACCTCCGTCCTGTTTAGG-3′ (forward) and 5′-CGAAGTCAGCTCCTTGGTTC-3′ (reverse), GAPDH 5′-AACGGATTTGGTCGTATTGG-3′ (forward) and 5′-TTTGGAGGGATCTCGCTCCT-3′ (reverse). To detect the expression of LCN2 in CAC cells, PCR amplification was performed for 30 cycles at 94°C for 30 s, 57°C for 1 min, and 72°C for 2 min using Taq polymerase. The amplified PCR products (10 μl) were separated on 2% agarose gels and stained with Redsafe™ (Intron, Daejeon, Korea). DNA band intensity was quantified using an NαBI imager (Neogene Science, Suwon, Korea).

### Small interfering RNA for inhibition of specific gene expression

Small interfering RNA (siRNA) sequences used for targeted silencing of the LCN2 (NCBI Ref Seq NM_005564.4), p65 (NCBI Ref Seq NM_001145138.1), and STAT3 (NCBI Ref Seq NM_003150.3) genes were from Ambion (Austin, TX, U.S.A.). Specific and Scrambled siRNA (Ambion, Austin, TX, U.S.A.) were transfected into the cells using TransiT-X2® transfection reagent (Mirus Bio, Madison, WI) according to the manufacturer’s protocol.

### Protein extraction and Western blotting

Cytoplasmic and nuclear protein extraction were prepared as previously described [29]. For whole cell extract, the cells were harvested by resolving in RIPA buffer (Thermo Fisher Scientific, Waltham, MA) and centrifuged at 13200 rpm at 4°C for 30 min. After centrifugation, supernatants were used as whole cell extracts and the concentration was measured using a Bradford Reagent (Sigma–Aldrich). Thirty to fifty micrograms of protein was loaded on to 8–12% of SDS/polyacrylamide gels. After transferring and blocking, each polyvinylidene difluoride (PVDF) membrane was probed with the primary antibodies to LCN2 (1:2000, AF1757, R&D Systems), p65 (1:1000, SC-8008 Santa Cruz Biotechnology), STAT3 (1:2000, #4904, Cell Signaling Technology), phospho-STAT3 (1:2000, #9131, Cell Signaling Technology), PI3K (1:1000, SC-7176, Santa Cruz Biotechnology), phospho-PI3K (1:2000, #4228, Cell Signaling Technology), mTOR (1:2000, #2972, Cell Signaling Technology), phospho-mTOR (1:2000, #2971, Cell Signaling Technology), AKT (1:2000, #9272, Cell Signaling Technology), phospho-AKT (1:2000, #4060, Cell Signaling Technology), XIAP (1:2000, #14334, Cell Signaling Technology), Bcl-2 (1:2000, #2872, Cell Signaling Technology), Bcl-*xL* (1:1000, SC-8392, Santa Cruz Biotechnology), Mcl-1 (1:2000, #4572, Cell Signaling Technology), COX-2 (1:1000, SC-1745, Santa Cruz Biotechnology), Lamin B (1:2000, SC-6216, Santa Cruz Biotechnology) and Actin (1:2000, A2066, Sigma–Aldrich). Immunoreactive bands were detected by using enhanced ECL prime (GE Healthcare, NJ, U.S.A.), captured by an Las-3000 luminescent Image Analyzer (FujiFilm, Tokyo, Japan).

### Cell transfection and dual-luciferase reporter assay

The pGL3-pLCN2 promoter was obtained from Addgene (Cambridge, MA). The amplified plasmid-DNA was confirmed by using restriction enzyme (BglII). The cells (1 × 10^5^ cells) were transfected with 500 ng of the pGL3-LCN2 reporter plasmid together with 2.5 ng of the internal control plasmid phRL-TK (*Renilla* luciferase vectors) using Lipofectamine™ 2000 (Invitrogen, U.S.A.) according to the manufacturer’s protocol. To analyze the responses to specific inhibition of NF-kB and STAT3, the cells were transfected with the pGL3-pLCN2 reporter plasmids, together with 2.5 ng of the internal control plasmid. After 24 h, the cells were stimulated with 50 ng/ml IL-6. For inhibition study using specific inhibitors, the cells were treated with specific inhibitors and/or IL-6 after transfection with pGL3-pLCN2/phRL-TK. Reporter plasmid and siRNA were transfected together for inhibition study using targeting siRNA followed by the addition of IL-6. Luciferase activity was measured using a Dual-Luciferase Reporter® assay system (Promega) by Lumat LB 9507 (Berthold, Bad Wildbad, Germany). The efficiency of transfection was normalized by using the *Renilla* luciferase activity derived from phRL-TK. The data shown are the mean ± standard error (SE) for independent triplicate samples.

### Statistical analyses

The data are presented as the mean ± SE of at least three independent experiments performed in duplicate. Representative blots are shown. All the data were entered into GraphPad Prism 5.0 was used to perform two-tailed *t* tests, Mann–Whitney tests, analysis of variance (ANOVA) or Kruskal–Wallis tests, where appropriate. A *P*-value <0.05 was considered significant.

## Results

### LCN2 is highly up-regulated in murine CAC models induced by AOM/DSS

**Figure 1:**
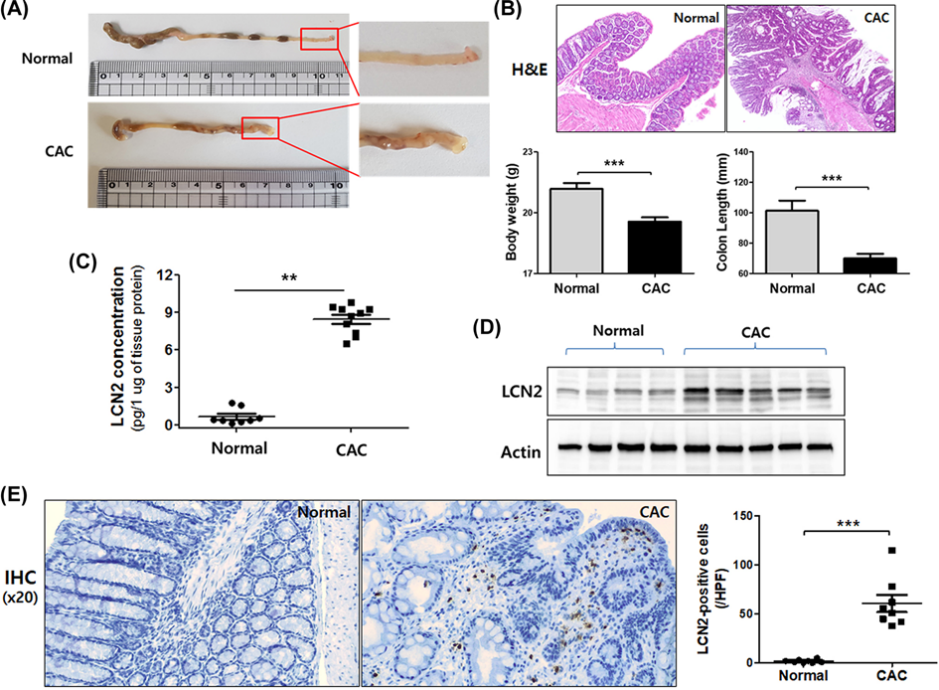
The expression of LCN2 was specifically induced in colon tissue of AOM/DSS-induced CAC mouse models The mice were given 7.4 mg/kg body weight of AOM and 3% DSS and subsequently killed (normal = 10, AOM/DSS = 12 mice). (**A**) Macroscopic appearance was captured for detecting tumor formation in the colon. (**B**) H&E staining (magnification ×10) of colonic mucosal tissue sections from mice were photographed using microscopy (upper panel). Colon length and body weight were statistically analyzed (lower panel). Data shown are presented as the mean ± SE. **P<0.01 versus control and CAC group. (**C**) After homogenizing colonic tissues, levels of LCN2 in tissue were detected by ELISA as described in the ‘Materials and methods’ section. Data shown are presented as the mean ± SE. **P<0.01 versus the control and CAC groups. (**D**) Total tissue extracts were prepared after killing and analyzed using LCN2 and actin antibody by Western blotting. Actin was used as a loading control. (**E**) IHC images of LCN2 expression in the colonic tissues from control and CAC mice are shown and the positively binding cells are shown in brown. The numbers of positive cells in the tumor samples were counted. Values represent means ± SEs. ***P<0.001 vs. the normal group.

Although LCN2 is involved in certain cases of IBD and CRC, its expression level in the CAC murine model is unclear. To determine expression levels of LCN2 in CAC, we applied AOM/DSS murine models. As shown in the representative images in Figure 1A, numerous nodular, polypoid, or caterpillar-like tumors were observed in the middle and distal colon of CAC models. Moreover, the results of H&E staining of colonic tissues from AOM/DSS-induced cancer models showed severe inflammatory lesions that exhibited total impairment of glandular structure, mucosal ulceration, crypt damage, and infiltration of immune cells. Changes in body weight and colon length are well-known characteristics of colon carcinogenesis. A significant reduction in body weight was observed in CAC group compared with that in control group. Colon length also showed significant shortening in the CAC group (Figure 1B).

We examined LCN2 concentration in colon lysates in animal models (Figure 1C). ELISA revealed that LCN2 levels were significantly higher in mice with CAC (0.698 ± 0.221 pg/1 μg of protein), a 12-fold increase when compared with normal colon (8.425 ± 0.353 pg/1 μg of protein). In addition, LCN2 protein expression levels in CAC colonic tissues were higher than in normal colonic tissues (Figure 1D).

To verify LCN2 expression in murine models, we performed IHC using paraffin-embedded colonic tissue. As shown in Figure 1E, more LCN2-positive cells were observed in the CAC group than in the normal control group and the number of positively stained cells was significantly higher in CAC mice than in normal mice. These results suggest that LCN2 might be involved in colon carcinogenesis from chronic inflammation.

### LCN2 is specifically up-regulated by IL-6 in human CRC cells

**Figure 2:**
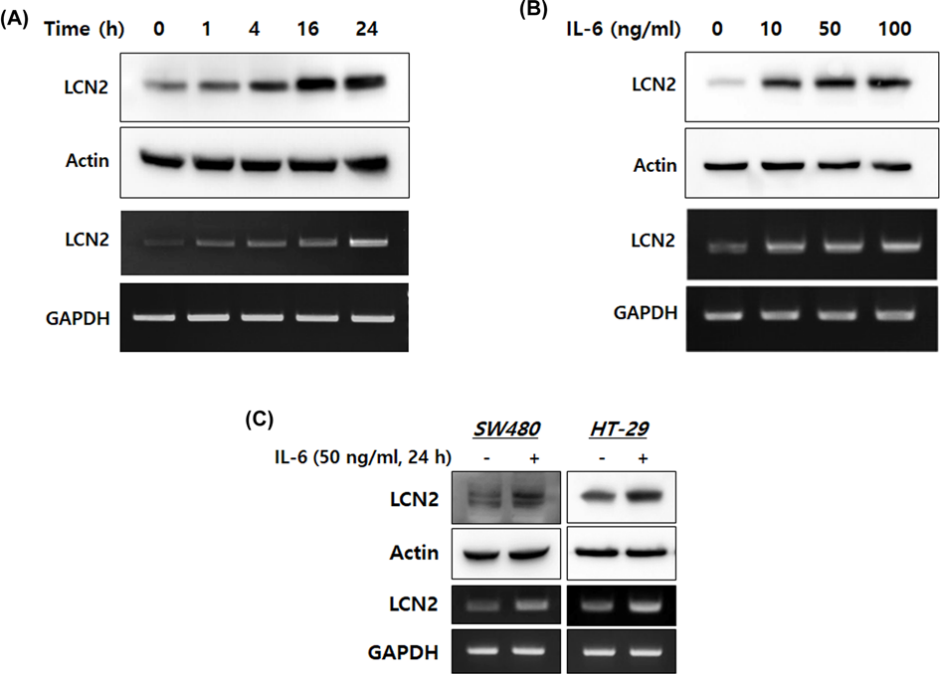
IL-6 specifically up-regulated LCN2 protein and mRNA levels in human CRC cells (**A**,**B**) After stimulation with IL-6, protein and mRNA of DLD-1 cells were isolated. Then, Western blotting and reverse-transcriptase PCR were performed. The top images are Western blots and lower images are reverse-transcriptase PCR. (**C**) Human CRC cell lines, SW480 and HT-29, were stimulated with 50 ng/ml IL-6 for 24 h, then the levels of LCN2 protein and mRNA were detected. All images are representatives from at least three independent experiments.

To investigate whether IL-6 could be responsible for the up-regulation of LCN2, we analyzed the expression of LCN2 mRNA and protein at different time points and IL-6 concentrations. After stimulation with 50 ng/ml IL-6, DLD-1 cells showed induction of LCN2 mRNA and protein in a time-dependent manner (Figure 2A); after 24 h, sustained dose-dependent increases in LCN2 mRNA and protein levels were also observed (Figure 2B). To confirm these findings in other CRC cells, we observed alterations in LCN2 levels on stimulation with IL-6 in SW480 and HT-29 cells. As shown in Figure 2C, we observed dramatic up-regulation of LCN2 protein and mRNA after 24 h of stimulation with IL-6 at a dose of 50 ng/ml. These results indicate that the IL-6 inflammatory mediator strongly induces LCN2 transcription and translation in human CRC cells.

### IL-6-specific up-regulation of LCN2 is dependent on STAT3 activation in human CRC cells

**Figure 3:**
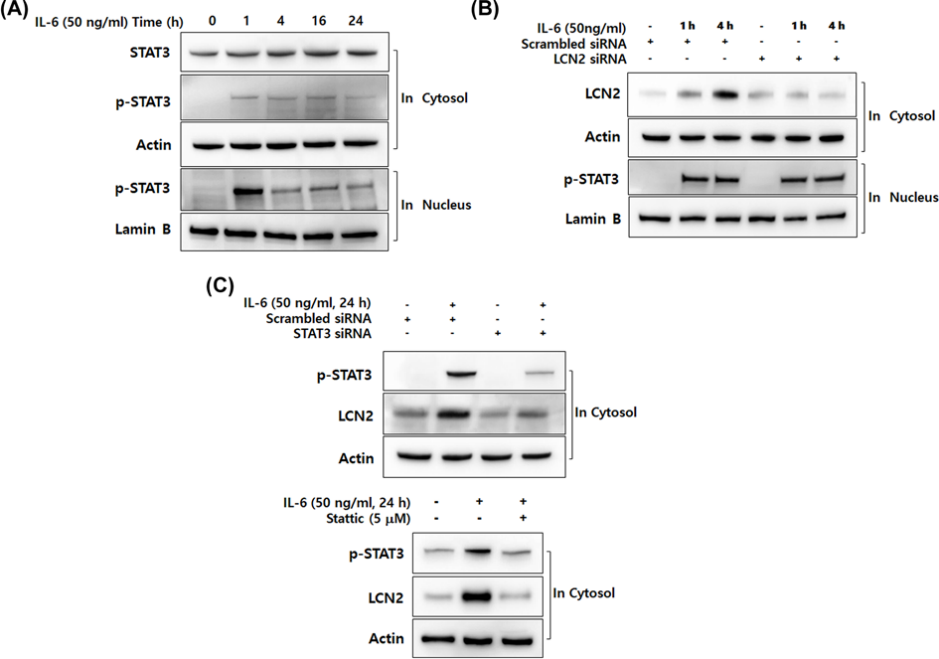
IL-6-specific up-regulation of LCN2 is dependent on STAT3 activation in human CRC cells (**A**) After stimulation with 50 ng/ml IL-6 for the indicated time, cytosolic and nuclear extracts of DLD-1 cells were prepared and used to determine the translocation of p-STAT3. Actin and lamin B were used as loading controls. (**B**) After transfection with LCN2 siRNA, DLD-1 cells were stimulated with 50 ng/ml IL-6. Then the level of LCN2 in cytosol and p-STAT3 in the nucleus were analyzed by Western blotting. Actin and lamin B were used as loading controls. (**C**) The cells were stimulated after transfection with STAT3 siRNA, then the level of LCN2 and p-STAT3 were analyzed by Western blotting (upper panel). After pretreatment with 5 μM stattic (STAT3-specific inhibitor), the cells were stimulated with IL-6. Then, the level of LCN2 and p-STAT3 were detected using Western blotting analysis (lower panel). Actin was used as a loading control. All images are representatives from at least three independent experiments.

IL-6 is a strong activator of STAT3, causing the activation and translocation of STAT3 signaling when combined with its receptor [30]. Therefore, we investigated whether up-regulation of LCN2 by IL-6 is involved in the activation of STAT3 in CRC cells. Western blot analysis was performed using DLD-1 cells at different time points. As shown in Figure 3A, IL-6 started to phosphorylate STAT3 after 1 h and stabilized until 24 h. Strong translocation of p-STAT3 to the nucleus was also observed after 1 h of stimulation with IL-6. Although nuclear translocation of p-STAT3 declined slightly, expression of p-STAT3 was weak until 24 h.

To clarify the molecular mechanism of LCN2 mediation by IL-6 in the STAT3 pathway, we examined whether LCN2 silencing affected IL-6-promoted STAT3 activation in CRC cells (Figure 3B). DLD-1 cells were transfected with scrambled and LCN2 siRNA, then the cells were stimulated by 50 ng/ml of IL-6 at peak time points of STAT3 activation (1 and 4 h). Interestingly, we did not observe any alteration of STAT3 activation between scrambled siRNA and LCN2 siRNA-transfected cells. To further investigate the role of STAT3 in IL-6-induced LCN2, STAT3 targeting siRNA and STAT3-specific inhibitor stattic were used in human CRC cells (Figure 3C). Transfection with STAT3 siRNA in DLD-1 cells diminished the expression of LCN2 induced by IL-6. When cells were pretreated with 5 μM of stattic, IL-6 induced LCN2 protein levels were also decreased by STAT3 inhibition. These results indicate that IL-6 induction of LCN2 is dependent on STAT3 activation in CRC cells.

### IL-6-specific up-regulation of LCN2 is also dependent on NF-κB activation in human CRC cells

**Figure 4:**
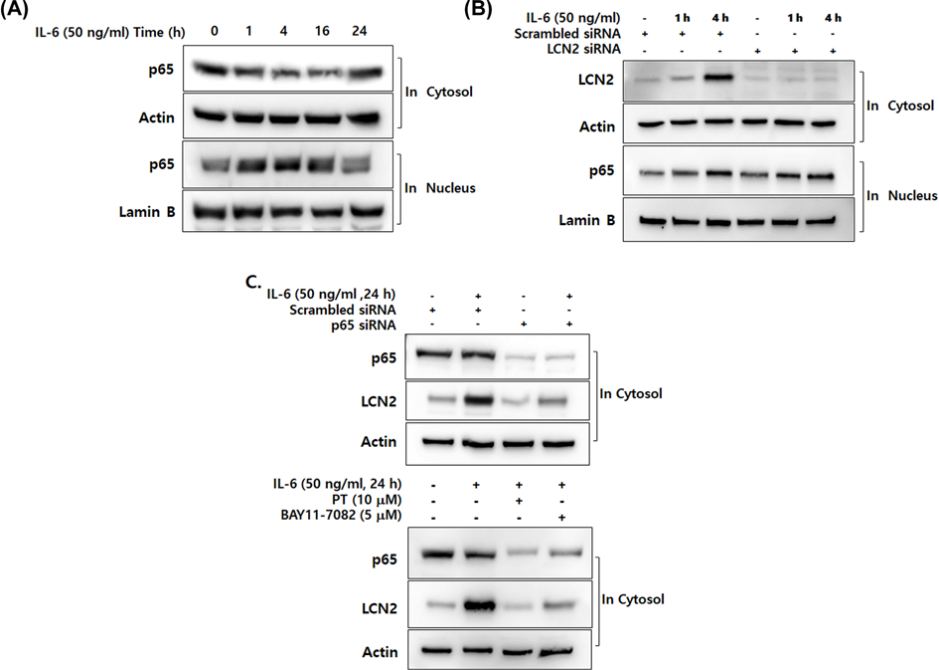
IL-6-specific up-regulation of LCN2 is dependent on NF-κB activation in human CRC cells (**A**) After stimulation with 50 ng/ml IL-6 for the indicated time, cytosolic and nuclear extracts of DLD-1 cells were prepared and used to determine the translocation of p65 from the cytosol to the nucleus. Actin and lamin B were used as loading controls. (**B**) After transfection with LCN2 siRNA, DLD-1 cells were stimulated with 50 ng/ml IL-6. Then the level of LCN2 in the cytosol and p65 in the nucleus were analyzed by Western blotting. Actin and lamin B were used as loading controls. (**C**) The cells were stimulated after transfection with p65 siRNA, then the levels of LCN2 and p65 were analyzed by Western blotting (upper panel). Cells were stimulated with IL-6 after pretreatment with PT and BAY11-7082 (NF-κB-specific inhibitor). Then, the levels of LCN2 and p65 were detected using Western blot analysis (lower panel). Actin was used as a loading control. All images are representatives from at least three independent experiments.

Since IL-6 is a potent proinflammatory cytokine and NF-κB activation is considered an essential mechanism of inflammation, we investigated whether IL-6-stimulated LCN2 is related to the NF-κB pathway in human CRC cells. In response to stimuli, NF-κB subunit p65 translocates to the nucleus followed by proteolytic degradation of IκB-α [31]. To determine whether NF-κB is activated by IL-6, we performed Western blot analysis using DLD-1 cells after stimulation with IL-6. As shown in Figure 4A, nuclear translocation of p65 was greatest at 4 h, but translocation decreased slightly thereafter.

To elucidate the molecular mechanism of LCN2 in NF-κB activation following stimulation with IL-6, we examined whether NF-κB activation is altered by LCN2 silencing (Figure 4B). After transfection with siRNA, DLD-1 cells were incubated with 50 ng/ml of IL-6 for 1 and 4 h to maximally stimulate NF-κB activation. As shown in Figure 4C, we observed that IL-6-promoted NF-κB activation is unaffected by LCN2. To validate whether NF-κB activation is required for IL-6-induced LCN2 in CRC cells, the cells were analyzed by Western blotting. IL-6-induced LCN2 was dramatically down-regulated in p65-transfected cells when compared with control cells. We also observed that treatment with PT and BAY11-7082, which are NF-κB-specific inhibitors, suppressed expression of LCN2 in IL-6-stimulated cells. These results suggest that IL-6 induction of LCN2 is dependent on NF-κB activation in CRC cells.

### IL-6 induces LCN2 promoter activity under control of the NF-κB and STAT3 signaling pathways

**Figure 5:**
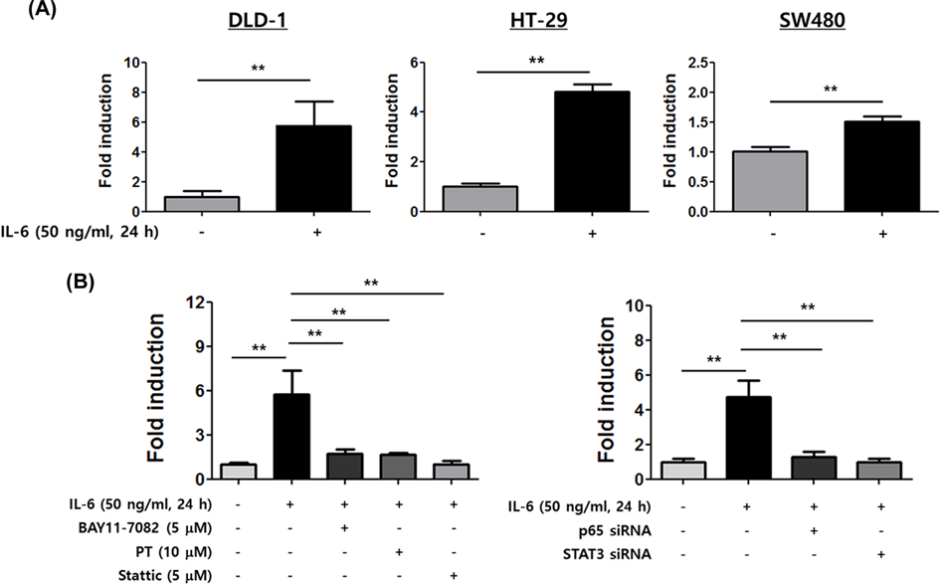
IL-6 specifically induces LCN2 promoter activity under control of the NF-κB and STAT3 signaling pathways (**A**) Human CRC cells were transfected with reporter plasmid carrying a 1233-bp fragment of the LCN2 promoter, then the growth medium supplemented with IL-6 was added to the cells. After 24 h, the cells were harvested and promoter activities were determined. The data represent the mean ± SE of three independent experiments. **P<0.01 versus unstimulated cells. (**B**) After transfection with LCN2 promoter construct, IL-6 and specific inhibitors were added to DLD-1 cells for 24 h. Then, the promoter activities of LCN2 were determined (left). DLD-1 cells were co-transfected with LCN2 promoter construct and indicated siRNA, then the cells were stimulated with IL-6 for 24 h. Alterations in LCN2 promoter activities were detected. The data represent the mean ± SE of three independent experiments **P<0.01 versus unstimulated cells or stimulated cells.

To further identify the regulatory mechanisms involved in up-regulation of LCN2, we transfected CRC cells with a 1233-bp LCN2 promoter construct that was subsequently stimulated with IL-6 (Figure 5A). Following stimulation with IL-6, a dramatic increase in LCN2 promoter activity was observed when DLD-1 and HT-29 cells were transfected with the LCN2 promoter-inserted vector (5.7-fold in DLD-1 cells and 4.8-fold in HT-29 cells). A slight induction in LCN2 promoter activity was observed in SW480 cells after stimulation with IL-6. These data support that stimulation of LCN2 by IL-6 occurs at the transcriptional level.

To determine whether STAT3 and NF-kB activation is required to induce the LCN2 promoter activity stimulated by IL-6, we examined the effect of specific inhibitors of NF-kB and STAT3 on DLD-1 cells transfected with LCN2 promoter construct (Figure 5B). As expected, specific inhibitors of NF-kB caused 3.5-fold (PT) and 3.3-fold (BAY11-4082) decreases in LCN2 promoter activity following IL-6 induction. Moreover, stattic, a STAT3 specific inhibitor, yielded a dramatic decrease in LCN2 promoter activity until 5.5-fold, very similar to the activity of unstimulated cells. The experiment using p65 and STAT3 siRNA also showed a significant reduction in LCN2 promoter activity following IL-6 stimulation compared with stimulated control cells (3.8-fold in p65 siRNA-transfected cells and 4.8-fold in STAT3 siRNA-transfected cells). This indicates that induction of both STAT3 and NF-kB activation by IL-6 allows up-regulation of LCN2 promoter activity.

### IL-6-induced LCN2 regulates cell survival and anti-apoptotic molecules mediated by the NF-kB/STAT3 pathway

**Figure 6:**
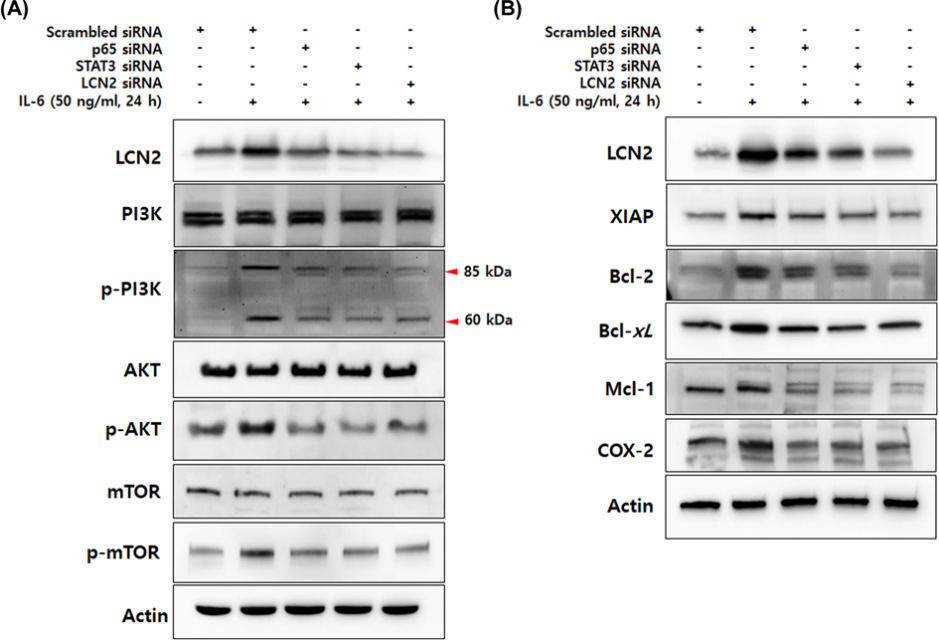
IL-6-induced LCN2 regulates cell survival and anti-apoptotic molecules mediated by the NF-kB/STAT3 pathway (**A**) After transfection with p65, STAT3 and LCN2 siRNA, DLD-1 cells were stimulated with 50 ng/ml IL-6 for 24 h, total extracts of DLD-1 cells were prepared and used to determine the phosphorylation of PI3k/AKT/mTOR. Actin was used as a loading control. (**B**) Total extracts prepared by the same methods were analyzed to determine the protein levels that are target gene products of STAT3 and NF-kB. Actin was used as a loading control. All images are representatives from at least three independent experiments.

To investigate the role of LCN2 in cancer development driven by chronic inflammation, we analyzed the molecular mechanisms associated with cell survival and apoptosis that are downstream of the NF-kB/STAT3 pathway by Western blotting. The PI3K/AKT/mTOR pathway, which plays an important role in survival and proliferation of cancer cells, is also activated by IL-6 [32]. Moreover, IL-6-induced JAK2/STAT3 is required for activation of the PI3K/AKT pathway via up-regulation of AKT1 promoter activity [33]. As expected, we observed an increase in phosphorylated PI3K, AKT, and mTOR on IL-6 stimulation. Interestingly, the stimulated cells showed a dramatic reduction in phosphorylated PI3K, AKT and mTOR when they were transfected with STAT3 siRNA as well as p65 and LCN2 siRNA (Figure 6A).

**Figure 7:**
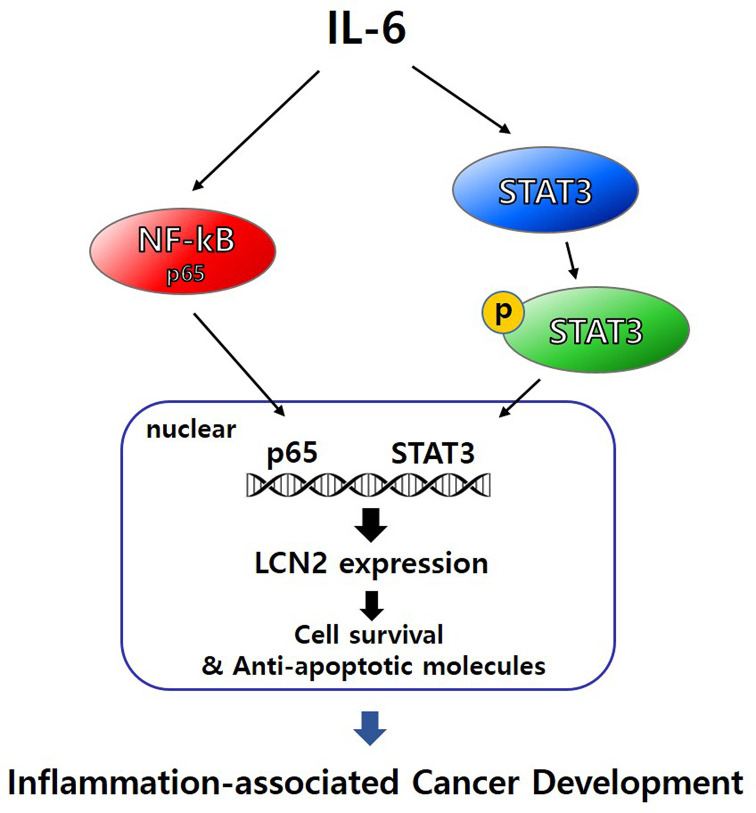
Schematic representation of the proposed mechanisms of IL-6-induced LCN2 via NF-kB and STAT3 pathway in inflammation-associated cancer development

To further delineate the underlying mechanisms, we explored the alteration of NF-kB and STAT3 target gene products when stimulated cells were transfected by targeting siRNA against p65, STAT3, and LCN2 (Figure 6B). The expression of anti-apoptotic proteins Bcl-2, Bcl-*xL*, XIAP, and Mcl-1 in stimulated cells was markedly reduced by silencing p65, STAT3, and LCN2. Moreover, survival and progression-related protein COX-2 was also dramatically suppressed by p65, STAT3, and LCN2-targeted siRNA. Taken together, these data suggest that LCN2 regulates protein expression related to cell survival and cancer progression mediated by NF-kB and STAT3 activation. The proposed molecular mechanisms of IL-6-induced LCN2 on colon cancer development are illustrated in Figure 7.

## Discussion

Chronic inflammation is a driving force behind cell transformation and malignant progression [34]. The contribution of chronic inflammation to carcinogenesis has been demonstrated in various types of cancer including liver cancer (from hepatitis B and C virus infection), gastric cancer (from *Helicobacter pylori* colonization), lung cancer (from smoking and asbestos exposure), and CRC (from UC and Crohn’s disease) [35]. During the chronic inflammation process, tumor-infiltrating immune cells produce cytokines that signal transformed cells and support their growth and survival [36,37]. IL-6, a pro-tumorigenic cytokine, stimulates STAT3 activation, and it has been demonstrated that IL-6-induced STAT3 activation is necessary for the development of CAC [8,10]. In the present study, we demonstrated that LCN2 plays a key role in carcinogenesis from intestinal inflammation and up-regulation of LCN2 is strongly dependent upon IL-6-induced STAT3 activation. To the best of our knowledge, there has been no prior investigation of LCN2 as a biomarker in CAC using experimental animal models, or of the underlying mechanisms of LCN2 in the STAT3 signaling pathway.

LCN2 was originally considered a biomarker of acute kidney injury due to its function in capturing bacterial siderophores [38]. Elevated levels of LCN2 mRNA and protein have been observed in various types of cancers including CRC, suggesting that LCN2 may serve as a biomarker for cancer. Although the functional role of LCN2 in cancer has been investigated, studies linking LCN2 to cancer as a driver of chronic inflammation are still lacking. In 2017, Gomez-Chou et al*.* demonstrated that LCN2 promotes pancreatic ductal adenocarcinoma (PDAC) by modulating the secretion of proinflammatory cytokines in human pancreatic cancer stellate cells, the first suggestion of a possible mechanism through which LCN2 contributes to the development of PDAC in the tumor microenvironment [39]. However, the underlying mechanism and functional role of LCN2 from the perspective of inflammation-associated cancer are still unclear. In the present study, we provided new insight into LCN2 as a potential biomarker for the prediction of CAC, and demonstrated that LCN2 is a key regulator of cell survival and tumor development mediated by STAT3/NF-kB activation.

Involvement of NF-kB signaling in inflammation and cell survival is well established, and persistent NF-kB activation in inflammation has been suggested to contribute to tumorigenesis [40,41]. Activated NF-kB was detected in lamina propria macrophages and epithelial cells from biopsy specimens or cultured cells from IBD patients as well as in tissue from CRC patients [42–44]. Anti-inflammatory therapy with nonsteroidal anti-inflammatory drugs inhibits the NF-kB signaling pathway and thereby reduces the risk of CAC by 75–81% [45]. Although the evidence for potential involvement of NF-kB in IBD and CRC is quite substantial, direct genetic proof of its role in tumor initiation by chronic inflammation did not exist until now. Our findings suggest that IL-6-induced up-regulation of LCN2 is strongly dependent upon NF-kB activation, and LCN2 is required for CRC cell survival mediated by NF-kB. Several publications demonstrated that proinflammatory cytokine-specific up-regulation of LCN2 is controlled by the NF-kB signaling pathway. In 2003, Cowland et al. found that IL-1β induces LCN2 expression in A549 lung adenocarcinoma cells at the transcriptional level by an NF-kB-dependent pathway and showed that IL-1β stimulation can induce binding of NF-kB to the LCN2 promoter [46]. In 2006, they also reported that IL-1β specificity is caused by the need for an LCN2 promoter in NF-kB-binding cofactor IkB-ζ for transcriptional activation in A549 cells [47]. Consistent with the results of Cowland et al., we demonstrated that induction of LCN2 promoter activity in human CRC cells is dependent upon NF-kB activation. In addition, we suggested a new mechanism of LCN2 in the tumor microenvironment through which IL-6, a powerful cytokine in CAC, is able to specifically increase LCN2 promoter activity under control of NF-kB activation.

STAT3 is a critical transcription factor for IL-6 signaling. A possible role for STAT3 in the development of CAC has been suggested by the finding that activation of STAT3 signaling is persistent in patients with IBD and CRC [48,49]. Thus, the IL-6/STAT3 signaling pathway has potential as a preventive and therapeutic target in patients with CAC. Here, we found that IL-6-induced LCN2 is inhibited by STAT3 siRNA and specific inhibitors, suggesting that LCN2 is a downstream target of STAT3 signaling. Moreover, we demonstrated that LCN2 promoter activity in CRC cells is regulated in a STAT3-dependent manner. LCN2 is regulated by STAT3 signaling. Only one previously published paper showed the relevance of STAT3 and LCN2 expression in response to inflammatory cytokines. Jung et al. reported that IL-10 specifically up-regulates LCN2 in primary human macrophages and LCN2 promoter activity is STAT3 dependent. They observed that LCN2 secreted from macrophages in response to IL-10 induces cellular growth and proliferation of MCF-7 breast cancer cells, suggesting that macrophage-derived LCN2s might contribute to tumor development and progression [50]. IL-10 was originally considered an anti-inflammatory cytokine, and its role in tumor pathogenesis and development is extremely controversial as it exhibits both anti-tumor and tumor promotion characteristics [51]. Therefore, our study extends and confirms the previously described results that induction of LCN2 in response to cytokines is dependent on STAT3 activation and LCN2 expression and contributes to tumorigenesis in the setting of chronic inflammation.

Once activated, NF-κB and STAT3 control the expression of survival, anti-apoptotic, pro-proliferative and immune response genes [52,53]. Some of these genes overlap and require transcriptional cooperation between NF-κB and STAT3 [54]. Thus, the positive feedback and collaboration between STAT3 and NF-κB play critical roles in controlling communication between inflammatory and cancer cells. To address the role of LCN2 in the collaboration between STAT3 and NF-kB activated by IL-6, we analyzed the expression of the STAT3-mediated PI3K signaling pathway and NF-kB/STAT3 target genes related to survival, anti-apoptosis, and tumor progression. The PI3k/AKT signaling pathway, which plays a critical role in cell survival, is phosphorylated by IL-6-induced STAT3/JAK2 activation [55]. Our results showed that down-regulation of p65 and LCN2 using specific siRNA led to inhibition of PI3K/AKT/mTOR phosphorylation by IL-6. We also observed that target gene products of NF-kB (Bcl-2, Bcl-*xL*, XIAP, and COX-2) and STAT3 (Bcl-2, Bcl-*xL*, and Mcl-1) are suppressed by transfection of p65, STAT3, and LCN2 siRNA. These results strongly suggest that NF-kB and STAT3 cooperatively regulate these downstream pathways or target gene products, and LCN2 is a capable intermediate between the two transcription factors.

Because of the high incidence and lethality of CRC cases worldwide, identifying novel regulatory pathways involved in CRC development and growth is critically important. The molecular mechanisms underlying the involvement of chronic inflammation in tumorigenesis are far from completely understood. Further insight into cancer-derived chronic inflammation will help us to develop effective cancer therapies or even prevention. Here we have presented novel findings on the role of LCN2 in CAC pathophysiology. LCN2 contributes to survival and cancer development in the tumor microenvironment. Molecular evidence suggests that regulation of LCN2 involves the NF-kB/STAT3 signaling pathway and LCN2 acts as an intermediary between NF-kB and STAT3. The proposed molecular mechanisms of IL-6-induced LCN2 in inflammation-associated cancer are illustrated in Figure 7.

Further animal studies using LCN2-knockout mice will be needed to determine the precise role of LCN2 in CAC. However, we propose that LCN2 expression in CRC may serve as a novel therapeutic target for the prevention and treatment of CAC.

## Data Availability

All data generated or analyzed during the present study are included in this published article.

## Abbreviations

AOM: azoxymethane
CAC: colitis-associated cancer
CRC: colorectal cancer
DMSO: dimethylsulfoxide
DSS: dextran sulfate sodium
H&E: Hematoxylin and Eosin
IBD: inflammatory bowel disease
IL: interleukin
LCN: lipocalin 2
NF-κB: nuclear factor-κB
PBS: phosphate-buffered saline
PDAC: pancreatic ductal adenocarcinoma
PT: parthenolide
SE: standard error
siRNA: small interfering RNA
STAT3: signal transducer and activator of transcription 3
